# Human *GLI3* Intragenic Conserved Non-Coding Sequences Are Tissue-Specific Enhancers

**DOI:** 10.1371/journal.pone.0000366

**Published:** 2007-04-11

**Authors:** Amir Ali Abbasi, Zissis Paparidis, Sajid Malik, Debbie K. Goode, Heather Callaway, Greg Elgar, Karl-Heinz Grzeschik

**Affiliations:** 1 Institute of Human Genetics, Philipps-University, Marburg, Germany; 2 School of Biological and Chemical Sciences, Queen Mary University of London, London, United Kingdom; Ecole Normale Supérieure de Lyon, France

## Abstract

The zinc-finger transcription factor GLI3 is a key regulator of development, acting as a primary transducer of Sonic hedgehog (*SHH*) signaling in a combinatorial context dependent fashion controlling multiple patterning steps in different tissues/organs. A tight temporal and spatial control of gene expression is indispensable, however, *cis*-acting sequence elements regulating *GLI3* expression have not yet been reported. We show that 11 ancient genomic DNA signatures, conserved from the pufferfish *Takifugu (Fugu) rubripes* to man, are distributed throughout the introns of human *GLI3.* They map within larger conserved non-coding elements (CNEs) that are found in the tetrapod lineage. Full length CNEs transiently transfected into human cell cultures acted as cell type specific enhancers of gene transcription. The regulatory potential of these elements is conserved and was exploited to direct tissue specific expression of a reporter gene in zebrafish embryos. Assays of deletion constructs revealed that the human-*Fugu* conserved sequences within the *GLI3* intronic CNEs were essential but not sufficient for full-scale transcriptional activation. The enhancer activity of the CNEs is determined by a combinatorial effect of a core sequence conserved between human and teleosts *(Fugu)* and flanking tetrapod-specific sequences, suggesting that successive clustering of sequences with regulatory potential around an ancient, highly conserved nucleus might be a possible mechanism for the evolution of cis-acting regulatory elements.

## Introduction

Mutations in the human transcription factor GLI3 cause a variety of dominant developmental defect syndromes, subsumed under the term “GLI3 morphopathies” [1], including Greig cephalopolysyndactyly syndrome (GCPS) [2]–[4], Pallister-Hall syndrome (PHS) [5], postaxial polydactyly type A (PAPA) [6], and preaxial polydactyly type IV (PPD-IV) [1]]. Mutations affecting murine *Gli3*, such as extra toes (*Xt*), anterior digit deformity (*add*), and polydactyly Nagoya (*Pdn*) serve as models for GLI3 morphopathies [7]–[10]. All GLI3 morphopathies show malformations of the autopod, i.e. polydactylies or syndactylies. In addition, craniofacial abnormalities are associated with GCPS, and in the most severe form, PHS, other developmental malformations occur, such as hypothalamic hamartoma, visceral anomalies, anus atresy, epiglottis and larynx defects [11].

Genotype-phenotype correlation has been reported for Pallister-Hall syndrome with mutations deleting the C-terminal part of GLI3, 3′of the zinc finger encoding domain, leaving the DNA-binding domain intact [12], [13]. Functional haploinsufficiency of *GLI3* appears to cause GCPS, since deletions or translocations eliminating one allele as well as missense or nonsense mutations distributed over the entire coding sequence are associated with this phenotype [2], [4], [12].

The transcription factor GLI3, together with its paralogues GLI1 and GLI2, acts as a primary transducer of Sonic hedgehog (SHH) signaling in a context dependent combinatorial fashion [14]. GLI3 and GLI2 can act both as transcriptional activators or repressors whereas GLI1, whose expression is transcriptionally regulated by GLI2 and GLI3, appears to play a secondary role in potentiating the SHH response [15]–[18]. In murine embryos, the locations affected in human GLI3 morphopathies, in particular the forebrain and the autopod, show strong *Gli3* expression [8]. In humans, a lower level of GLI3 in these locations due to haploinsufficiency is inadequate for normal development. Apparently, the amount of gene product produced by one *GLI3* allele is sufficient in most other locations.

Mouse embryos with homozygous *Gli3* deficiency show pleiotropic and lethal congenital malformations with distinct preaxial limb polydactylies [8], [9]. A multitude of studies in mice and other model organisms have proven that a GLI-code, the interplay of GLI proteins and the temporally fine tuned expression of the *GLI* genes in adjacent domains, together provide a basic tool that is used over and over again in embryonal development. This is applicable to patterning along the anteroposterior axis [19], induction of sclerotome [20], morphogenesis of the neural tube [21], [14], [22], the prosencephalon [23], and cerebellum [24], anterior-posterior limb patterning [25], chondrocyte differentiation [26], skeletal muscle formation [27] and prostate gland development [28]. These data indicate that GLI3 has essential functions controlling multiple patterning steps in different tissues/organs, and therefore a tight temporal and spatial control of gene expression is indispensable.

The identification of *cis*-acting regulatory elements interacting with the *GLI3* promoter could facilitate the detection of factors controlling the tissue specific availability of GLI3 *in trans* in Hedgehog (HH) target cells. In turn, identification of transcription factors for spatial and temporal control of *GLI3* expression would greatly enhance our understanding of the regulatory network that coordinates the multitude of patterning events associated with the HH signaling pathway. Mammalian enhancers can be defined by a combinatorial code for an assembly of transcription factor binding sites (TFBS), but *in silico* identification has proven difficult. This is firstly due to the paucity of information about TF binding specificity, confined to a set of loose consensus binding motifs. Secondly, transcription factors generally recognize only six to eight base-pair DNA motifs, and the distance over which they may be located around a particular gene could be vast [29]. Enhancer elements have been observed at a distance of more than a megabase from their target gene [30]. To narrow the sequence intervals to be scrutinized experimentally for *cis*-acting regulatory potential, multispecies highly conserved non-coding sequences (CNEs) have been targeted [31]. CNEs are much more conserved than the sequences of known enhancers, but many of these elements clearly regulate gene expression [32]. They also might play a role in other processes, e.g. as repressors, replication origins or modulators of chromatin structure. The reason for the strong evolutionary constraint over extended lengths of DNA sequence is not known. Sequence conservation of *cis*-regulatory elements of transcription within CNEs might date back to the period in evolution when the new patterns that they determine were added to a basic body plan. Non-coding sequence elements conserved from *Fugu* to man might harbour enhancers directing a basic outline common to the two distantly related vertebrates, whereas tetrapod specific CNEs might only contain regulatory elements for later additions to the body plan, such as an autopod with digits.

As an initial attempt to identify and characterize the regulatory code directing human *GLI3* expression, we have applied reporter gene assays to test the regulatory potential of 11 intronic *Fugu*-human CNEs in cultured cells with or without endogenous *GLI3* expression. All elements are able to regulate expression in a cell type dependent fashion. The elements identified as potential enhancers extend beyond the *Fugu*-human highly conserved core sequences into flanking, less well conserved DNA. These core sequences are necessary but not sufficient for full regulatory potential. By expressing reporter genes under the control of the human *GLI3*-CNEs in zebrafish embryos, we demonstrate that the activating or repressor potential of CNEs observed in human cell culture transient transfection assays is retained *in vivo* in a teleost fish. Enhancers with activating potential differ in their tissue specificity, however, none of them direct expression exclusively in one tissue. Nevertheless, to a large extent reporter gene expression patterns mimic endogenous zebrafish *gli3* expression. We conclude that human-*Fugu* CNEs, located in the introns of *GLI3,* mark critical components of the cis-regulatory inventory for temporal and spatial expression control of this key developmental gene.

## Results

### *GLI3* Tetrapod-Teleost Conserved Non-coding Elements (CNEs) are located exclusively within introns

**Figure 1:**
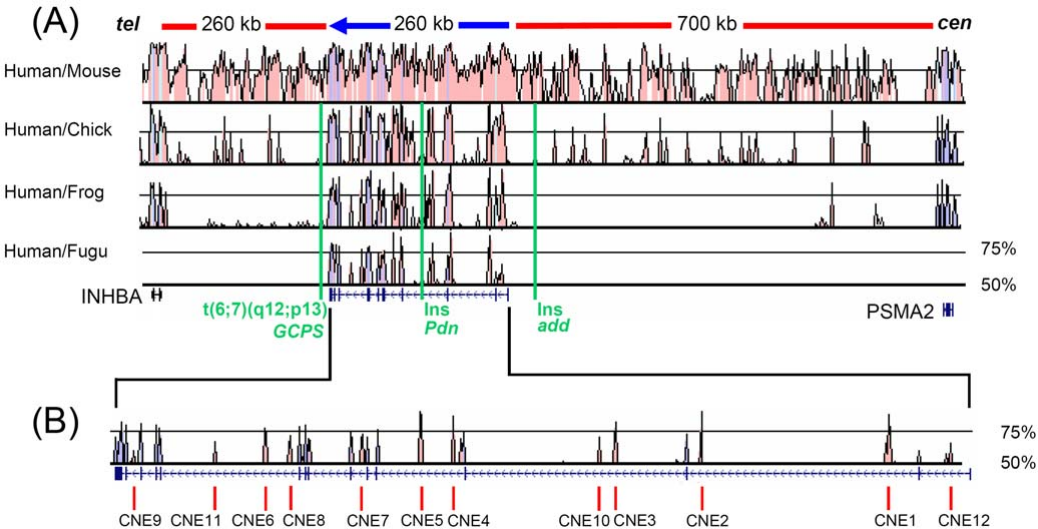
Comparative Sequence Analysis of the *GLI3* Locus Detects Conserved Non-coding Sequence Elements. (A) Sequence alignments of the genomic interval containing the human *GLI3* locus and flanking human genes *INHBA* and *PSMA2* with orthologous counterparts from representative members of rodent, bird, amphibian, and fish lineages. These are shown as SLAGAN derived VISTA representations. Conserved coding sequences are depicted in blue and conserved non-coding sequences are in pink. Criteria of alignment were 60 bp window and 50% conservation cutoff. Conservation between human and *Fugu* (scaffold_210 ENSEMBL genome browser) is restricted to the *GLI3* gene. Red bars above the conservation plot depict the approximate length of intergenic regions flanking human *GLI3.* The blue arrow shows the length of the *GLI3* gene and the direction of transcription. A graphic representation showing exons and introns of *GLI3* is shown below the homology plot. Green vertical lines indicate the positions of alterations affecting the genomic structure of the locus which result in loss of GLI3 function: a translocation event associated with Greig cephalopolysyndactyly syndrome (GCPS) [36], and two insertions (ins) in mouse mutants anterior digit pattern deformity (*add*) [7], [37] and polydactyly Nagoya (*Pdn*) [38]. (B) Magnified view of the human/*Fugu* conservation plot and the genomic structure of human *GLI3*. The red vertical bars below the plot show the position of human/*Fugu* highly conserved non-coding sequence elements (CNEs) that were functionally tested as putative enhancers.

The pufferfish *gli3* (scaffold_210; ENSEMBL genome browser) is tightly bordered by genes that are not orthologous to the human *GLI3* flanking regions. Therefore, it is more likely that non-coding sequences conserved between human and *Fugu* and which might be potential enhancers, are restricted to *GLI3* introns. *GLI3* is flanked by variable gene desert [33]. Comparison of approximately 1 Mb human genomic DNA sequence encompassing *GLI3* and extending up to the flanking genes with the complete assembly of the *Takifugu rubripes* genome sequence indicates that sequence homology is restricted to the gene region proper (Figure 1).

Multi-species alignment of *GLI3* genomic sequences from mammals revealed extensive conservation, which obscured the identification of potentially functional elements embedded in intronic DNA (Figure 1A). However, in the transition from moderate (mammalian sequence comparison) to intermediate evolutionary distance (human vs birds/amphibia) the extent of neutrally evolving sequences dropped sharply, whilst sequence comparison at an extreme phylogenetic distance (human/teleost) reduced the number of candidates further. This allowed us to prioritize 11 CNEs for functional analysis.

These CNEs are distributed across almost the entire *GLI3* interval (Figure 1B), with 2 elements in each of introns 2, 3, 4, and 10 and one in each of introns 1, 6, and 13. The amplicons encompassing ancient signatures within flanking sequences highly conserved only in mammals are described in Table 1. CNE2 represents an ultraconserved element [34] (>200 bp at 100% identity in human, rat and mouse) and will be described elsewhere. A further element, CNE8, located in intron 10 has not yet been tested functionally. Using both extrinsic and *ab initio* approaches embedded at the UCSC browser and the Ensembl gene build system, we found no evidence for overlap with putative protein coding regions or non-coding RNA genes. In each of the 11 CNEs we predict transcription factor binding sites (TFBSs) for established developmental regulators (Table 1) using the programs Consite and rVista v 2.

**Table 1:** Tetrapod-Teleost Conserved Non-Coding elements (CNEs) from Introns of Human *GLI3* Selected for Functional Analysis

Region	Element	Amplicon Coordinates Chr7	Amplicon Size	Conservation Human-Fugu 50%; >60 bp	In Vitro Activity	In Vivo Activity	Conserved Putative TFBSs
Intron 1	CNE12	42239221-42239879	659 bp	190 bp	A/R	n.a.	TBX5, PITX2, PAX6, GATA1, POU6F1
Intron 2	CNE1	42219598-42220542	945 bp	935 bp	A/R	(+)	ATF1, CDPCR1, CDXA, EBOX, FOXM1, FOXP3, GABP, GATA1, PBX1, HOXA3, LMO2COM, MSX1, MYOGENIN, NFY, NMYC, POU3F2, USF, YY1, IRF1, AFP1, VJUN, dHAND
Intron 2	CNE2	42159050-42159483	434 bp	401 bp	n.a.	n.a.	CEBPDELTA, CHCH, HOX13, IRF2, LEF1B, MSX1, SP3, TCF4, EN1
Intron 3	CNE3	42131347-42131748	400 bp	378 bp	R	(−)	AREB6, ATF, EBOX, GATA1, GATA2, GATA3, LEF1B, LMO2COM, MYOD, NMYC, TCF4, USF
Intron 3	CNE10	42125837-42126969	1133 bp	105 bp	A/R	(+)	CART1, CDP, CLOX, P53, E2F1, SOX5, EN1, PBX1
Intron 4	CNE4	42079507-42079678	172 bp	160 bp	R	(−)	CREL, LEF1B, NKX25, PTF1BETA, STAT1, STAT4, STAT6
Intron 4	CNE5	42068665-42069242	578 bp	255 bp	A/R	(−)	AREB6, E2F, FREAC2, GATA1, GATA6, HNF1, HNF3 ALPHA, MEIS1, OCT1, PAX2, PBX1, PBX, TBP, XFD1
Intron 6	CNE7	42049418-42050221	804 bp	337 bp	A/R	(+)	NKX61, OCT1, POU3F2, SRY, MEF2, STAF
Intron 10	CNE8	n.a.	n.a.	123 bp	n.a.	n.a.	n.a
Intron 10	CNE6	42018164-42019025	862 bp	179 bp	A/R	(+)	OCT1, PPARA, TBX5, PBX1, PAX4
Intron 10	CNE11	42002211-42003395	1185 bp	129 bp	A/R	(+)	SMAD3, LEF1B
Intron 13	CNE9	41975857-41976525	669 bp	108 bp	A/R	(+)	*OCT1, PPARA, TBX5, PAX3, STAT5A*

Location, size, coordinates (NCBI 36, Oct 2005), and human-*Fugu* conserved transcriptional factor binding sites (union of results from rVISTA and ConSite) are indicated. Dual nature and repressor elements are represented by “A/R” (activator/repressor) and “R” symbols, respectively. The (+) sign indicates the elements which induced GFP expression in zebrafish embryos, while (−) sign indicates those which could not drive GFP expression significantly. n.a.: not analyzed. The analysis of CNE2 is reported elsewhere.

### Cell Based Reporter Assays Reveal a Context Dependent Dual Nature (Activator/Repressor) of CNEs

**Figure 2:**
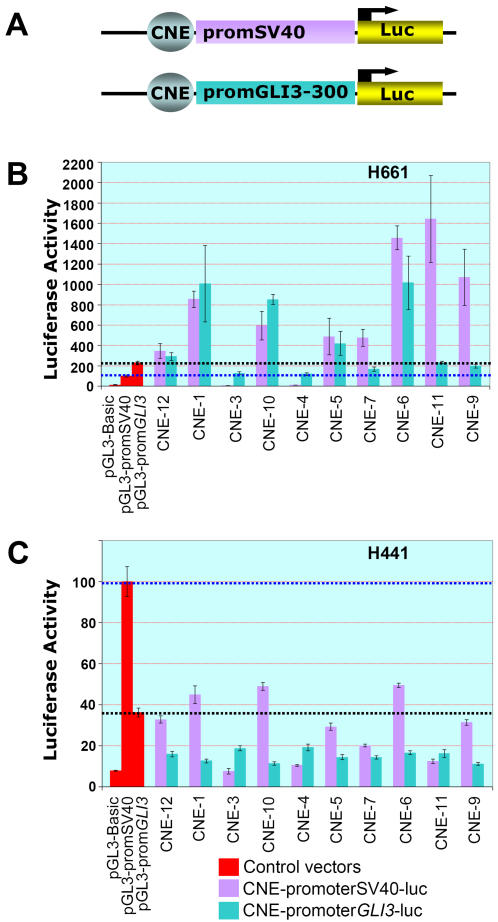
CNEs Regulate Luciferase Reporter Gene Expression in Transiently Transfected Human Cell Lines. (A) Diagrams of the reporter constructs employed to test the regulatory potential of CNEs from the introns of human *GLI3*. CNEs were cloned into in a pGL3-Basic vector containing either a minimal *GLI3* promoter (pGL3-CNE-prom*GLI3*-300-luc) or a heterologous SV40 promoter (pGL3-CNE promSV40-luc) upstream of a firefly luciferase gene. (B) Luciferase activity of reporter constructs in human H661 cells that express endogenous *GLI3*. (C) Luciferase activity of reporter constructs in human H441 cells that do not express endogenous *GLI3*. The pGL3-Basic vector, with no promoter/enhancer insert was used as a negative control. Luciferase activity in cells transiently transfected with the positive control, a construct containing a SV40 promoter upstream of the reporter gene (pGL3-promSV40-luc), was taken as 100% (blue dotted line). A plasmid expressing *Renilla* luciferase was co-transfected as a standard for transcription efficiency. Average firefly luciferase reporter activities relative to *Renilla* luciferase activity from three triplicate transfection experiments are depicted as percentage of activity obtained with the positive control vector (B, C). Standard errors of the mean are shown. Black dotted lines indicate the luciferase expression level reached in each cell line with the pGL3-prom*GLI3*-300-luc vector.

In order to test the selected subset of 10 sequence elements for their potential to regulate reporter gene expression, recombinant constructs with CNEs placed in either orientation upstream of a luciferase gene controlled by either the heterologous SV40 promoter or the human minimal *GLI3* promoter (Figure 2A), were transiently transfected into two human kidney fibroblast lines. The H661 cell line expresses endogenous *GLI3* whereas H441 does not express this gene (data not shown). In dual luciferase assays eight elements (CNE 1, 5, 6, 7, 9, 10, 11, and 12) showed activating potential in H661 cells whereas two elements (CNE3 and CNE4) repressed reporter gene expression below the level achieved by either promoter alone (Figure 2B).

In contrast, when tested in the H441 cell line, all CNEs exhibited a strong repressing activity (Figure 2C). Thus, the cell based reporter assay identified two categories of intra-*GLI3* regulatory elements: firstly context independent repressors and secondly enhancers with a context dependent dual nature, serving as activators in a GLI3 positive context and as repressors in cells without endogenous *GLI3* expression.

### *In Vivo* Functional Analysis of CNEs with Transiently Transfected Zebrafish Embryos

The CNEs that have been tested *in vitro* were next tested *in vivo* using zebrafish as a model organism. CNEs were co-injected with a GFP reporter into zebrafish embryos and then monitored for enhancer activity at set time points.

**Figure 3:**
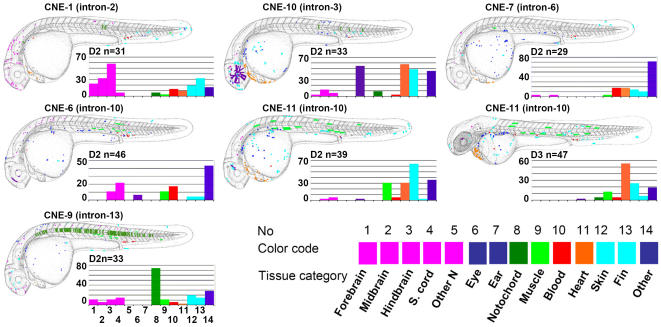
Sites of GFP Expression Induced by *GLI3*-Associated CNEs in Zebrafish Embryos. Upregulation of GFP by individual *GLI3*-associated CNEs (indicated by name and location in a *GLI3*-intron) depicted in schematic representations of day two, 24–33 hpf (D2) or day 3 (D3) embryos. N = the total number of positive embryos per CNE. Categories of cell type that were positive for a given element are color coded, and each dot represents a single GFP positive cell. These are mapped onto camera lucida drawings of the zebrafish embryo, and the overall results are overlaid. The percentage of positive embryos that show expression in each color coded tissue category are shown on the bar charts.

**Figure 4:**
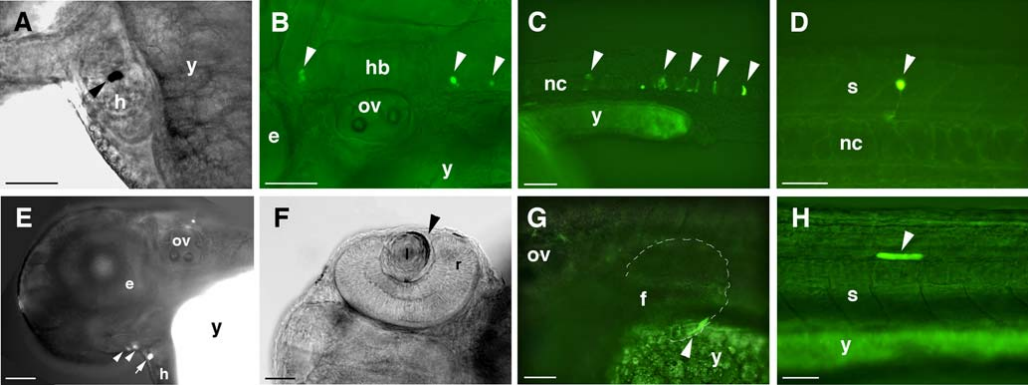
Tissue Type Specific Expression of GFP Reporter Gene in Zebrafish Embryos. Examples of GFP expression induced by CNEs 1, 9, 10, and 11 are shown in fixed tissues after wholemount anti-GFP immunostaining (bright field views A and F) or in live embryos by combined bright field and GFP fluorescence microscopy analyses (B, C, D, E, G and H). Arrowheads indicate GFP expressing cells. Embryos C and D are ∼26–33 hpf, while embryos A, B, E, F, G, and H are 48–54 hpf. Lateral views, anterior to the left and dorsal to the top except for F where the dorsal view is shown. GFP positive cells were found in the following: (A) CNE1, heart chamber (B) CNE1, hindbrain neurons (C) CNE9, notochord (D) CNE9, spinal cord neuron (E) CNE10, lower jaw primordia and pericardial regions (F) CNE10, lens epithelial cell layer (G) CNE11, pectoral fin (H) CNE11, muscle. (e) Eye; (f) fin; (h) heart; (hb) hindbrain; (I) lens; (nc) notochord; (ov) otic vesicle; (r) retina; (s) spinal cord; (y) yolk.

With the exception of CNE5, the *in vitro* identified cellular context dependent enhancer elements drove GFP expression in significant proportion of microinjected zebrafish embryos (Figures 3 and 4), whereas neither CNE3 nor CNE4 could induce reporter gene expression in fish embryos. At day two of development (∼26–33 hours post fertilization, hpf), CNE1 directs GFP expression prominently in various subdivisions of CNS, forebrain, midbrain and hindbrain with 22%, 32% and 58% of expressing embryos respectively. Within the cardiac chambers, GFP expression induced by CNE1 was observed in 10% of expressing embryos at day two and in 30% on day 3 of development (∼50–54 hpf). Reporter gene expression was also observed in blood cells of day 2 embryos (12%) skin (19%) and developing median fin fold (32%).

CNE10 directs reporter gene expression most frequently in eye (54% of expressing embryos), pericardial region (57%) and skin cells (48%). Within the eye, CNE10 mediated reporter expression in retinal ganglion cells, the photoreceptor layer at the retinal margin, the lens epithelial cell layer and the lens nuclear region. CNE10 also induces GFP expression in the lower jaw primordia or first pharyngeal arch (mandibular arch) region in significant proportion (24%) of day 2 (∼26–33 hpf) expressing embryos.

CNE7 did induce reporter gene expression in different regions of day 2 embryos (∼26–33 hpf) but the activity was not particularly strong in any one tissue/region of the embryo.

CNE6 drove GFP expression most prominently in the spinal cord neurons (21% of expressing embryos), and less frequently in hindbrain neurons immediately flanking the hindbrain/spinal cord boundary (10%), in blood cells (17%), and muscle fibers (10%).

CNE11 activity on day 2 of development was confined to skin cells (64% of expressing embryos), muscle fibers (30%) and heart (30%). In contrast to other elements, which drove expression mainly on day 2 (∼26–33 hpf) of development, CNE11 also strongly enhanced reporter expression on day 3 (50–54 hpf) of development (Figure 3), within heart chambers (55%) skin cells (25%), muscle fibers (12%), with some expression in the pectoral fins.

A particularly prominent GFP expression domain for CNE9 injected embryos was in notochord cells (74% of GFP expressing embryos). In addition, reporter gene expression occurred in spinal cord (14%), forebrain (11%), hindbrain (11%), skin cells (20%), fin (14%), and muscle fibers (11%).

### *In Vitro* Deletion Analysis of Selected Sub-set of CNEs

**Figure 5:**
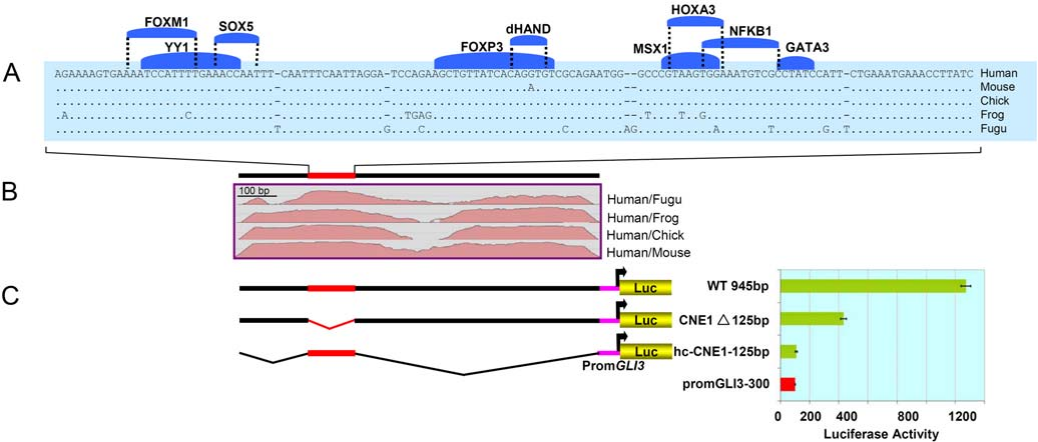
Deletion Analysis Reveals a Critical Role of *hc-CNE1-125bp* for the Regulatory Potential of CNE1. (A) BLASTZ alignment of a human, mouse, chick, frog, and *Fugu* highly-conserved 125 bp sequence fragment embedded within CNE1 shown with predicted conserved TFBSs (above). (B) SLAGAN alignment plots of human, mouse, chick, frog and *Fugu* CNE1 using human sequence as the base line. (C) Architecture of CNE1 wild type and deletion constructs, The red bar depicts the highly conserved region, and less well conserved regions are shown in black. Luciferase activity obtained in H661 cells after transient transfection of reporter constructs is shown in the diagram at the right side. Reporter gene expression is driven by CNE1 fragments upstream of the human *GLI3* minimal promoter. The red bar depicts luciferase expression (100%) in H661 cells driven alone by the control *GLI3* minimal promoter (Prom-*GLI3*-300), while green bars represent the activity recorded for the vectors containing experimental reporter constructs.

In order to define functionally critical regions within CNEs and to understand the significance of strength of evolutionary constraints on defining their overall activity, we prioritized three elements CNE1, CNE5, and CNE6 for dissection and subsequent analysis of the fragments by transient transfection assays in H661 cells. CNE1 spans a 945 bp human/fish conserved track with overall human/fish sequence similarity of ∼71%. Close inspection of CNE1 revealed a sequence block of ∼125 bp (*hcCNE1-125bp*) under particularly strong negative selection, almost unaltered in human/mouse and human/chick sequence comparisons, whilst a human/*Fugu* comparison shows ∼92% sequence identity (Figure 5A).

In order to test the functional significance of the *hcCNE1-125bp* track we generated two different deletion constructs. One contained *hcCNE1-125bp* alone with a minimal *GLI3* promoter. In the second construct, to investigate the activity of the moderately conserved human/*Fugu* flanking region, the *hcCNE1-125bp* fragment was deleted from the wild type CNE1(Figure 5B).

Deletion of the 125 bp highly conserved region reduced the activity by 67% compared to wild type construct, but it was still able to induce reporter gene expression 4-fold compared to the control vector, in which luciferase expression was driven alone by the minimal *GLI3* promoter. Tested alone, *hcCNE1-125bp* was unable to show any activating potential (Figure 5C).

**Figure 6:**
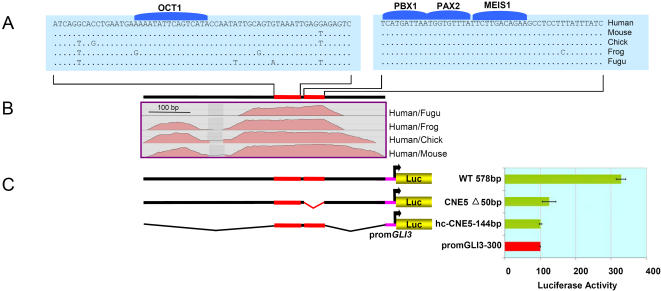
Putative Binding Sites for Individual Trans-Acting Factors are Necessary but not Sufficient for Activating Potential of CNE5. (A) BLASTZ alignment of highly conserved fragments embedded within CNE5 along with predicted conserved TFBSs. (B) CNE5 alignment plot of human, mouse, chick, frog and *Fugu* sequences using human sequence as the base line. (C) Architecture of wild type and deletion constructs; the red portion of the bar depicts the highly conserved human/fish regions. Luciferase activity obtained in H661cells after transient transfection of reporter constructs is shown in the diagram at the right side. Reporter gene expression is driven by CNE5 fragments upstream of the human *GLI3* minimal promoter. The red bar depicts luciferase expression (100%) in H661 cells driven alone by the control *GLI3* minimal promoter (Prom*GLI3*-300), while the green bars represent the activity recorded for the vectors containing experimental reporter constructs, i.e. wild type CNE5 (wt 578bp), CNE5 with deleted *PBX1, PAX2* and *MEIS1* binding module (*CNE5Δ50bp*), and the 144 bp fragment (*hcCNE5-144bp*). Deletion of the 50 bp fragment almost entirely extinguishes the strong activating potential of CNE5. The isolated 144 bp fragment cannot activate expression.

CNE5 harbors two highly conserved blocks interrupted by a short less well-conserved fragment (Figure 6B). Within one of these blocks, 100% conserved in multispecies sequence comparison from human to fish, phylogenetic footprinting reveals contiguous binding sites for three developmentally important homeobox and paired box transcription factors, *PBX1, PAX2* and *MEIS1*. The *PAX2* and *MEIS1* binding sites overlap by one nucleotide.

A 50 bp module encompassing the *PBX1, PAX2*, and *MEIS1* binding sites was excised from the wild type CNE5 fragment. Also, a 144 bp region encompassing both highly conserved blocks was isolated. Each of these were compared to wt CNE5 for their potential to enhance reporter gene transcription when transiently transfected into H661 cells. In contrast to wt CNE5, both elements were unable to activate basic transcription (Figure 6B and 6C).

**Figure 7:**
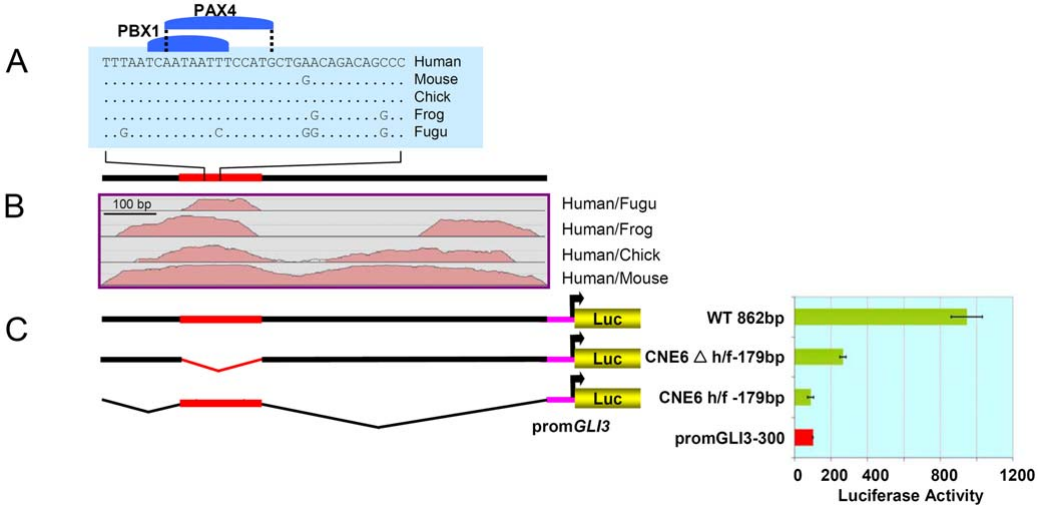
CNE6 Sequences Flanking Human/Fish conserved Track Show Residual Enhancer Activity. (A) BLASTZ alignment of the highest conserved 35 bp along with two predicted conserved TFBSs from the human/*Fugu* conserved block within CNE6. (B) CNE6 alignment plot of human, mouse, chick, frog and *Fugu* sequences using human sequence as the base line. (C) Architecture of wild type and deletion constructs; the red bar depicts the highly conserved human/fish segment. Luciferase activity obtained in H661 cells after transient transfection of reporter constructs is shown in the diagram at the right side. Reporter gene expression is driven by CNE5 fragments upstream of the human *GLI3* minimal promoter. The red bar depicts luciferase expression (100%) in H661 cells driven alone by the control *GLI3* minimal promoter (Prom*GLI3*-300), whilst the green bars represent the activity recorded for the vectors containing experimental reporter constructs, i.e. wild type CNE6 (wt 862bp), CNE6 with deleted human/*Fugu* conserved block (*CNE6Δh/f-179bp*), and the 72% human/fish conserved fragment (*CNE6h/f-179bp*). *CNE6Δh/f-179bp* can still enhance reporter gene transcription more than two-fold. The isolated 179 bp fragment cannot activate expression.

The wild type 862 bp CNE6 fragment shows 87% sequence identity in a human/mouse comparison and contains a 179 bp moderately conserved region between human/*Fugu*. This track encompasses a 35 bp highly conserved site (Figure 7A and 7B). In order to investigate the role of both the 179 bp human/fish region and the flanking tetrapod conserved elements in the overall *in vitro* enhancer activity of wt CNE6, each region was investigated separately (Figure 7C).

Deletion of the 179 bp element reduced the activity of CNE6 by ∼70% compared to the wild type construct. However, compared to the control vector, this deleted CNE6 was still able to up-regulate the reporter gene expression by more than 2-fold. The 179 bp fragment upstream of the minimal *GLI3* promoter did not result in up-regulation of reporter gene expression compared to the control (Figure 7B and 7C).

## Discussion

### Tetrapod-Teleost Conserved CNEs within Introns of *GLI3* Identify Enhancers

Human *GLI3* extends over 260 kb on chromosome 7p14.1 (Figure 1A), a gene poor region, and is flanked by ∼260 kb and ∼700 kb intergenic intervals [35]. *GLI3*-regulatory elements, potentially, could be located anywhere in this region. Considering observations with other developmental genes in gene poor regions, such as sonic hedgehog (*SHH*) [30], enhancers could even map within or beyond the neighbouring genes. In humans, the occurrence of distant regulatory elements can be heralded by cytogenetically detected translocations in patients with developmental malformations, causing the trait via separation of enhancer elements from their respective gene. In the case of *GLI3*, a translocation t(6;7)(q27;p13) truncating chromosome 7p14 about 10 kb downstream of the last exon results in a GCPS phenotype characteristic for functional deficiency of one *GLI3* allele [36] (Figure 1). Silencing of the intact *GLI3* gene in this case could be caused by loss of cis-regulatory sequences distal to the breakpoint. In the mouse, a transgene insertion ∼64 kb upstream of *Gli3* is associated with the phenotype of *anterior digit deformity* (add) [7], [37] (Figure 1). Here, the function of murine *Gli3*-enhancers located upstream of the insertion site may be disturbed. Thus, it is possible that *GLI3* might be among the genes regulated by distant enhancers. A further *Gli3* allele, the mouse mutant *Pdn*, results from insertion of a transposon into intron 3 (Figure 1) [38]. In those mice *Gli3* expression appears to be possible, though at a reduced level. Based on these observations, *GLI3* regulatory elements might be found up- or downstream of the gene or within the introns.

Algorithms for the prediction of enhancers determining the temporal and spatial expression of human genes are increasingly powerful [39], however, sound predictions of *GLI3* regulatory signatures have not yet been reported.

The region around *GLI3* is prohibitively large for using the painstaking strategy of stepwise deletions in reporter gene assays. Recently, it has been reported that there is a considerable overlap between experimentally verified enhancer elements and non-coding sequence elements (CNEs), that are evolutionarily conserved between distantly related species such as humans and the pufferfish [32]. This suggests that CNEs around or within a gene are promising candidates for enhancers of expression. Different levels of stringency have been applied for the definition of CNEs [34], [40], mostly with the intention to select a manageable number of candidate elements rather than with a biologically based rationale.

By employing multispecies sequence alignment we identified an ancient (tetrapod-teleost conserved) non-coding architecture within the introns of *GLI3*. The ancient, human/fish conserved signatures are embedded in larger sequence domains conserved in evolutionarily more recent species such as frog, chick or mouse (Figure 1). To test possible enhancers of expression we chose human/mouse CNEs encompassing >60 bp tracks with more than 50% sequence similarity between human and *fugu*. These candidate elements represent sequence that is under ancient, strong evolutionary constraint operating to maintain a DNA sequence signature.

### *In Vitro* Regulatory Activity of Intra-*GLI3* CNEs is Cell Type Specific

The majority of intra-*GLI3* CNEs (8/10) exhibited a cellular context dependent dual nature. In the endogenous *GLI3* expressing environment (H661) they functioned as activators whilst in the GLI3 negative (H441) cellular context they actively repressed the transcription (Figure 2). This differential activity is strong evidence in favor of assigning *GLI3*-specific regulatory potential to these CNEs. Similar context dependent dual-nature regulatory activity is known for other transcription factors [41]. Our *in vitro* investigation also revealed two of the CNEs that had a repressing potential even in a GLI3 positive cellular context.

The most plausible scenario to explain the dual nature of a sub-set of intra-*GLI3* enhancers could be the interaction of each CNE with different subsets of trans-acting factors (either activators or repressors of transcription) in a cellular context dependent manner [42], whilst elements with repressing potential, even in a GLI3 positive context, suggest the existence of context independent regulation.

### *In Vitro* Deletion Analysis Defines Functional Modules Within CNE1, 5 and 6

We tested a subset of the CNEs, each associated with unique sequence features, as potential enhancers in transient transfection assays in H661 cells to see if the core elements conserved in human-*Fugu* represent functionally critical regulatory modules. CNE1 spans a human/*Fugu* conserved region of exceptionally extended length, 935 bp, and embedded within it a highly constraint interval of 125 bp almost 100% conserved down to chick, while depicting a 92% conservation in human/fish comparison. CNE5 encompasses human to fish 100% conserved contiguous binding sites for developmentally important TFs PBX1, PAX2 and MEIS1. CNE6 docks a small moderately conserved human/fish track of 179bp, within human/mouse 862 bp track with overall 87% conservation.

Considering the known degeneracy of transcription factor binding target sites [43], the high conservation of the 125 bp track within CNE1 (Figure 5) over the course of such an extreme phylogenetic separation (human/fish) is unexpected [44]. A previous *in vivo* attempt to resolve the significance of a tetrapod-conserved non-coding sequence element encompassing a human-*Fugu* conserved region around the human *DACH* gene had revealed that alone, neither a human-*Fugu* conserved core nor the less conserved flanking region, functioned as activators [45]. The authors concluded that either the assay was not sufficiently sensitive or the core element might have an unknown biological function. In contrast, our *in vitro* deletion analysis with CNE1 provides evidence in favor of a quantitative participation of the conserved core to the overall activity of the enhancer, and suggests that this module embedded within CNE1 is essentially gene regulatory in function (Figure 5). The fact that the 125 bp sequence was unable to show any detectable activity in isolation reflects that this module is essential but not sufficient to uphold enhancer function on its own.

The manipulation of CNE5 (Figure 6) revealed that the deleted highly conserved element encompassing predicted contiguous TFBSs for developmental regulators *PBX1, PAX2* and *MEIS1* is necessary but not sufficient for the activating potential of this site.

The *in vitro* reporter gene expression data from deletion constructs of CNE6 suggest the existence of tetrapod specific functional constraints in the vicinity of an ancient fish specific element (Figure 7). It would appear that the overall enhancer activity is determined by the combinatorial affect of the ancient and the more recent sequences.

In all 3 examples, the excised elements had no activating potential when analyzed without the flanking sequences. However, we conclude from the sizeable reduction in activating potential in the absence of the core, that the human-*Fugu* conserved modules within the *GLI3*-CNEs are essential for transcriptional regulation. Mutagenesis of the predicted binding sites could show if transcription factors are involved in this function. The fact that flanking intervals of the human-*Fugu* conserved sequence elements contribute to the activity of the element, suggests that after the divergence of tetrapod-teleost lineages (450 Million years ago) there was a progressive gain of novel function centred around an ancient enhancer element. This possibly allowed fine-tuning of gene expression differentially in the tetrapod lineage, congruent with their complex developmental and anatomical needs.

### Can Transcription Factor Binding Sites Within CNEs Explain Their Evolutionary Conservation?

A possible restraint causing the maintenance of CNEs involved in gene regulation throughout vertebrates could be a strict combinatorial code of TFBSs where order and distance are critical. We have screened for intra-CNE human-*Fugu* conserved putative TFBSs using the computer programs Consite and rVista v 2. In order to increase the sensitivity and to reduce the number of false positives, we have combined the TFBSs motif searches with phylogenetic footprinting of CNEs across distantly related species [46], [47]. In each of the 11 sequences we identified human-*Fugu* conserved TFBSs for a number of developmental regulators (Table. 1). The prediction of binding sites for established developmental regulators under the highly stringent criteria in each of the tetrapod-teleost conserved intra-*GLI3* sequence tracks corroborates the conclusion from our experiments that the ancient elements contribute to the activity of the enhancer. However, TFBSs are known to allow considerable degeneracy and their overall density across each individual CNE is low. Unless strict maintenance of a combination of specific TFBSs and flanking sequence is required to retain tissue specifity of enhancer action, these sites may not contribute to the major constraint responsible for conservation of non-coding elements throughout evolution.

### Intra-*GLI3* CNEs Show Tissue Specific Regulatory Activity *In Vivo*

In order to address the *in vivo* role of *GLI3* associated conserved non-coding elements we selected a medium throughput strategy [31], employing transient reporter gene expression from the human *β*-globin promoter under the influence of a putative enhancer element in zebrafish embryos. This approach exploiting the transparency and rapid development of zebrafish embryos has recently shown its immense potential for functionally testing enhancer elements among conserved non-coding regions [31], [48]–[50]. Our results (Figure 3) indicate that the regulatory potential of most of the human CNEs defined in transient transfection assays of human cell cultures is similarly present in fish embryos. There is also a correlation between both enhancer and repressor activity *in vitro* and *in vivo*. Thus, we present evidence that both the sequence and the regulatory characteristics of *cis*-acting elements are conserved throughout evolution, from teleosts to man.

In mouse, GLI3 plays a prominent role in development of brain, ear, eye, craniofacial structures, limb and lung, and is also expressed in heart, kidney, skeletal muscles, fetal blood cells, epidermal cell layer of skin and other tissues (Mouse Genome Informatics http://www.informatics.jax.org). Zebrafish *gli3* is reported to be expressed in brain, dorsal spinal cord neurons, eye, and pectoral fin bud (Zebrafish Information Network; http://zfin.org) [51], [52]. However, exhaustive expression patterns throughout different stages of development have not been published.

A number of the positions in which transgene expression is observed coincide with known sites of GLI3 activity. For example CNE1 drives GFP expression predominantly in various subdivisions of the CNS, CNE10 activity was most frequent in the eye, pericardial region, lower jaw primordia and skin cells, CNE6 activity was more specific to hindbrain/spinal cord boundary neurons, muscle fibers and blood cells, and CNE11 driven reporter expression was largely restricted to cardiac chambers, skin cells and muscle fibers. Interestingly, CNE11 also induced GFP expression with low frequency within pectoral fins at day 3 of development which is consistent with the reported timing of zebrafish *gli3* expression in this tissue [51]. It can be seen that functional redundancy with respect to the site of expression was evident for all regulatory elements, a notion concordant with findings in other genes [53].

Some cell populations such as heart, the pericardial region, blood cells, muscle fibers, skin, and lower jaw primordial are domains of *Gli3* expression in mouse but not so far described in zebrafish. However, GLI3 functions appear to be conserved in mouse and zebrafish [51]. Therefore, the expression of g*li3* in zebrafish might be more extensive than reported so far. We observed expression in domains of the embryo where *gli3* is expressed neither in zebrafish nor in mouse. For example, CNE9 directed expression predominantly to the notochord, which is inconsistent with the reported endogenous *GLI3* expression in either species. This could reflect position effects upon the reporter-transgene inducing its expression at ectopic sites. The unexpected finding of a CNE within *GLI3*, which directs reporter gene expression at a site where GLI3 itself is never observed, stresses the importance of genomic context for the function of regulatory elements, as had been concluded by previous studies [31], [53], [54]. We must therefore exercise caution when trying to draw conclusions on the normal regulatory potential of genomic fragments based on reporter construct studies, in both cell culture and transgenic animals.

### Conserved Regulatory Elements are Uncovered by Sequence Comparison at Extreme Phylogenetic Separation

Most locations of reporter gene expression induced in transgenic zebrafish embryos by the human intronic *GLI3* CNEs represent prominent sites reported for endogenous zebrafish *gli3* [51]. However, zebrafish *gli3* expression in the pectoral fin bud has been reported to begin around 37 hpf, and by 44 h is expressed uniformly throughout the fin bud [51]. At this location only CNE 11 evoked signals in the pectoral fin (Figure 3), unlike the other enhancers, most of which ceased to act after ∼28–33 hrs. It is possible that more focused analysis may reveal additional expression in the fin bud, but most probably the array of potential cis-acting regulatory elements chosen in this study did not cover the complete toolbox of elements required to orchestrate *gli3* expression during zebrafish development. We have pinpointed the regions to be analyzed as potential enhancers by the presence of a human-*Fugu* conserved sequence element, but the extent of the fragments included as CNEs was defined from human/mouse comparison. By this approach we addressed an ancient core as well sequences flanking each human-*Fugu*-conserved element, which may have evolved in tetrapods after its divergence from the teleost lineage. It is of note that these flanking sequences show little identity in teleost genomes, yet still function as enhancers in zebrafish. Homology among non-coding intra-*GLI3* sequences of tetrapods is not restricted to areas identified through comparison with *Fugu*. CNEs uncovered by sequence comparison within tetrapods could form a rich source of further regulatory elements patterning tetrapod-specific additions to the body plan. It will be interesting to test if and where enhancers regulating expression of more modern structures, such as digits, direct reporter expression in the fish.

## Materials and Methods

### Reporter constructs

Candidate enhancer sequences (CNEs, Table 1) were PCR amplified using the high fidelity herculase enhanced DNA polymerase (Stratagene, Amsterdam, The Netherlands) with primers containing KpnI restriction site tags. Amplified DNA was purified using the QIAquick PCR purification kit (Qiagen, Hilden, Germany). Purified PCR products were then subjected to restriction site digestion with KpnI (New England Bio Labs, Ipswich, USA) and subsequently cloned in both orientations upstream of a minimal *GLI3* promoter or a heterologous SV40 promoter driving expression of the luciferase gene in the vector pGL3 (Promega, Madison, USA). The reporter constracts were designated pGL3-promGLI3-300-luc and pGL3-promSV40-luc, respectively. Recombinant reporter expression constructs were transfected into Top10 competent bacterial cells (Invitrogen, Karlsruhe, Germany) and subsequently isolated and purified using the Qiagen plasmid purification kit (Qiagen). To control the clones for presence of any point mutations generated during PCR amplification, appropriate DNA preparations were sequenced in ABI 377 automated sequencer (Applied Biosystems, Foster City, USA) and were analyzed with Sequencer software, Version 4.2.

### Deletion Mutants

The deletion mutants of selected CNEs were made by PCR using the recombinant reporter construct of each of the respective wild type CNE as a template. The sequences flanking the segment to be deleted were PCR amplified with two different sets of primers. One member of each set was wt primer tagged with a KpnI restriction site, while the other member was designed from the immediate vicinity of the sequence to be deleted and tagged with a HindIII restriction site. Amplified products flanking the region to be deleted were purified using the QIAquick PCR purification kit (Qiagen) and digested by HindIII then subsequently ligated to one another. The ligated products were size fractioned on 2% agarose gel, and the DNA fragment of expected length was gel excised, purified by using a QIAquick gel extraction kit (Qiagen), digested by KpnI, and inserted into the pGL3-promGLI3-300-luc reporter plasmid. Sequence of each deleted recombinant construct was confirmed by sequencing (ABI 377 automated sequencer; Applied Biosystems). In order to avoid the *de-novo* creation of transcription factor binding sites, compared to wild type sequence, each of the deleted sequences were analyzed for potential TFBS with the TESS web tool (Transcription Element Search Software on http://www.cbil.upenn.edu/tess).

### Cell Cultures

The human lung tumor cell line H661 and the human bronchiolar epithelial cells H441 were obtained from the ATTC, USA, and grown under standard conditions in RPM1-1640 medium (Sigma Aldrich, Missouri, USA) containing 10% fetal calf serum, 1% non-essential amino acids, 2% penicillin/streptomycin and 1% L-glutamine (H661) or in modified RPMI-1640 medium (Sigma Aldrich) with 25mM HEPES and sodium bicarbonate, containing 4% fetal calf serum, 1% non-essential amino acids, 2% penicillin/streptomycin and 1% L-glutamine (H441), respectively.

### Transient Transfection and Dual Luciferase Assay

The day before transfection, 4×10^5^ H661 or 3×10^5^ H441 cells were seeded into each well of a 12-well plate in 2 ml of the appropriate growth medium containing serum and antibiotics. After 24 hours of incubation at normal growth conditions, cells were transfected by using Effectene (Qiagen) according to the manufacturer's recommendations with the experimental firefly luciferase reporter constructs at a concentration of 200 ng/well, along with 100 ng/well of pRLSV40 (Promega) an expression vector containing cDNA encoding *Renilla* luciferase as an internal control reporter, and 200 ng/well of pGKBT7 (Clontech, Mountain View, USA) as a stuffer/carrier DNA.

48 hours after transfection, cells were assayed for luciferase activity with with the Dual-Luciferase Reporter Assay System (Promega) on an AutoLumat LB 953 luminometer (Berthold, Pforzheim, Germany). The activities of experimental reporter (firefly luciferase) were normalized to the activities of internal control reporter (*Renilla* luciferase). Triplicate assays were conducted three times.

### Zebrafish Enhancer/GFP Reporter Assay

Zebrafish were bred and raised according to standard protocols [55]. CNEs for co-injection were either cut out from plasmids or amplified by PCR and then purified by QIAquick PCR purification kit (Qiagen). The reporter expression construct consisting of cDNA encoding enhanced green fluorescent protein (EGFP) under the control of minimal promoter from the human, β-globin gene was also PCR amplified from plasmid construct (Clontech). Element DNA (250–300 ng/ul) and reporter DNA fragment (25 ng/ul) were combined with tracer, i.e. phenol red (0.1%), and co-injected into the embryos produced from natural mating with a femtojet pressure injection system (Eppendorf, Hamburg, Germany) at the 1- to 8-cell stage, embryos developing abnormally were discarded after 2 to 3 hours of injection. Normal embryos were raised in 0.003% phenylthiocarbamide in embryo medium from tailbud stage. On the second day of microinjection (approximately 26–33 hpf) embryos were dechorionated using pronase E, anaesthetized in Tricaine and analysed under UV-light for GFP expression by using an IX81 motorised inverted microscope (Olympus, Tokyo, Japan). Images were captured using an FVII CCD monochrome digital camera and analySIS image-processing software.

GFP expressing cells were classified according to the following tissue categories: forebrain, midbrain, hindbrain, spinal cord, eye, ear, notochord, muscle, blood (circulating)/blood islands, heart/pericardial region, epidermis and fins. GFP expressing cells that were not localized unequivocally were classified as others. Location and tissue category of each GFP-expressing cell for each embryo was recorded schematically using Adobe Photoshop software (Adobe Systems, San Jose, USA), onto an overlay of a camera lucida drawing of 31-hpf embryo. For each CNE, the GFP expression data was collected from 20-50 expressing embryos. As a control, mean of 200 embryos were injected with conserved coding and non-conserved intronic sequences along with the reporter system and were found unable to show any significant GFP induction.

Combined schematised expression data for each CNE was compressed into a JPEG file and coupled with graphical depiction of expression domains to present an overall impression of the spatial pattern to which the element directs expression.

### Anti-GFP Immunostaining

For immunostaining embryos were fixed in 4% paraformaldehyde overnight at 4°C and incubated with rabbit polyclonal anti-GFP (AMS Biotechnology, Abingdon Oxon, UK) using standard protocols [56] and the ABC amplification system (Vectastain; Vector laboratories, Burlingame, USA). Stained embryos were subsequently cleared in glycerol, flatmounted, and observed under bright field with Olympus IX81 motorised inverted microscope.

### Comparative Sequence Analysis

Approximately 1 Mb of the human genome, encompassing *GLI*3 (ENSG00000106571) as well as *GLI3* orthologous sequences of mouse (ENSMUSG00000021318), chick (ENSGALG00000012329), frog (ENSXETG00000001856) and *Fugu* (SINFRUG00000153715) were obtained from Ensembl genome browser (http://www.ensembl.org). Multispecies sequence comparison was performed by using the glocal alignment program Shuffle-LAGAN [57]. Human sequence was used as the baseline and annotated by using the exon/intron information available at Ensembl genome browser. Shuffle-LAGAN alignment was visualised with the VISTA visualization program [58]. The alignment was performed using 60 bp window and a cutoff score of 50% identity.

### *In Silico* Mapping of Conserved Transcription Factor Binding Sites

Human-*Fugu* conserved transcription factor binding sites in each CNE were detected with ConSite (http:/www.phylofoot.org/consite) and rVISTA.2.0 (http:/rvista.decode.org/). The ConSite screen for conserved TFBS was performed against the JASPAR database with 50% conservation cuttoff, 60 bp window size and 75% transcription factor score threshold settings.

rVISTA 2.0 searches for conserved TFBSs were performed against 500 vertebrate TF matrices from the TRANSFAC library, with matrix similarity cuttoff 0.85 by submitting a BLASTZ alignment file for each CNE to the rVISTA 2.0 site.
